# Sciatic–Vagal Nerve Stimulation by Electroacupuncture Alleviates Inflammatory Arthritis in Lyme Disease-Susceptible C3H Mice

**DOI:** 10.3389/fimmu.2022.930287

**Published:** 2022-07-18

**Authors:** Lavoisier Akoolo, Vitomir Djokic, Sandra C. Rocha, Luis Ulloa, Nikhat Parveen

**Affiliations:** ^1^ Department of Microbiology, Biochemistry and Molecular Genetics, Rutgers New Jersey Medical School, Newark, NJ, United States; ^2^ Center of Perioperative Organ Protection, Department of Anesthesiology, Duke University, Durham, NC, United States

**Keywords:** *Borrelia burgdorferi*, Lyme arthritis, inflammation, vagus nerve, sciatic nerve, electroacupuncture

## Abstract

Lyme disease is caused by *Borrelia burgdorferi*, and the pathogenesis of the disease is complex with both bacterial and host factors contributing to inflammatory responses. Lyme disease affects different organs including joints and results in arthritis. Immune responses stimulated by *B. burgdorferi* through toll-like receptors cause infiltration of leukocytes, which produce inflammatory cytokines and facilitate spirochete clearance. However, arthritic manifestations and chronic fatigue syndrome-like symptoms persist long after completion of antibiotic treatment regimens in a significant number of patients. To counter the effects of inflammation, treatment by non-steroidal anti-inflammatory drugs, hydroxychloroquine, or synovectomy to eradicate inflammatory arthritis in the involved joint could be employed; however, they often have long-term consequences. Acupuncture has been used for a long time in Asian medicine to diminish pain during various ailments, but the effects and its mechanism are just beginning to be explored. Control of inflammation by neuronal stimulation has been exploited as a systemic therapeutic intervention to arrest inflammatory processes. Our objective was to determine whether activation of the sciatic–vagal network by electroacupuncture on ST36 acupoint, which is used to control systemic inflammation in experimental models of infectious disorders such as endotoxemia, can also alleviate Lyme arthritis symptoms in mice. This aim was further strengthened by the reports that sciatic–vagal neuronal network stimulation can lead to dopamine production in the adrenal medulla and moderate the production of inflammatory factors. We first assessed whether electroacupuncture affects spirochete colonization to attenuate Lyme arthritis. Interestingly, bioluminescent *B. burgdorferi* burden detected by live imaging and qPCR were similar in electroacupuncture- and mock-treated mice, while electroacupuncture induced a lasting anti-inflammatory effect on mice. Despite the discontinuation of treatment at 2 weeks, the simultaneous decrease in neutrophils in the joints and inflammatory cytokine levels throughout the body at 4 weeks suggests a systemic and persistent effect of electroacupuncture that attenuates Lyme arthritis. Our results suggest that electroacupuncture-mediated anti-inflammatory responses could offer promising healthcare benefits in patients suffering from long-term Lyme disease manifestations.

## Introduction

Lyme disease affects nearly half a million people in the United States every year (1) and is the most prevalent tick-borne infection in North America and Europe. It is caused by the spirochete, *Borrelia burgdorferi sensu lato*, and is transmitted primarily by blacklegged deer tick, *Ixodes scapularis*, in the United States, while other *Ixodes* species are involved in transmission around the world (2). Ticks acquire the infection from the blood of infected hosts including the white-footed mouse, *Peromyscus leucopus*, which serves as the primary reservoir host, although other small animals can also serve as reservoirs for Lyme spirochetes. Typical early symptoms of Lyme disease are fever, headache, fatigue, and skin rash, called erythema migrans. Treatment with antibiotics in the early stages can generally clear infection and resolve disease symptoms; however, antibiotic refractory cases are often associated with persistent inflammatory arthritis, musculoskeletal aches, and neuronal syndrome (3–5). *B. burgdorferi* triggers protective host immune responses that include inflammatory responses in the affected tissues, which often persist for long periods of time even after clearance of the infection in humans (6). The primary innate immune responses include the activation of toll-like receptors (TLRs) by pathogen-associated molecular patterns (PAMPs) to stimulate the host defense against *B. burgdorferi* infection, but unregulated inflammation also worsens disease severity. Abundant lipoproteins on Lyme spirochete surface activate TLR2 signaling, which induces inflammatory responses to control spirochetes in the joints and mitigate *B. burgdorferi* infection, but these inflammatory factors can also enhance arthritis severity (7–10). Inflammatory reaction due to the release of *B. burgdorferi* DNA and RNA further activates the nuclear translocation of NF-κB and enhances the production of inflammatory cytokines and their downstream innate and adaptive immune responses. Thus, in addition to TLR2, *B. burgdorferi*-derived RNA activation of TLR8 and bacterial DNA activation of TLR9 in monocytes/peripheral blood mononuclear cells (PBMCs) induce type I interferon (IFN) and IFN-responsive host genes that contribute to Lyme arthritis (11, 12). Early in the infection, the presence of spirochetes and associated PAMPs increase innate immune responses and Th1 and Th17 cytokines, which tend to control the infection. Innate and Th1 cytokines decline markedly during convalescence, but Th17 cytokines fade slowly and may be the main mediators of antibiotic refractory arthritis and posttreatment Lyme disease syndrome (PTLDS) (13). Supporting this premise, the administration of antibodies against IL-17 and IL-23 delayed the onset of arthritis in C57/BL6 Lyme arthritis mouse models (14).

Lymphoid cells, particularly different types of T helper cells, affect arthritis severity. Murine and human lymphocytes express dopamine D2-like receptors, which can diminish inflammatory arthritic manifestations caused by the imbalance in Th17/activated Treg cells (15). Transforming growth factor β (TGF-β) plays a critical role in maintaining the balance of Th17/Treg cells such that TGF-β with IL-6 or IL-23 promotes differentiation of Th17 cells, whereas TGF-β with IL-2 induces differentiation of Tregs (16, 17). Furthermore, TLR2 stimulation in antigen-specific T cells has been shown to induce their proliferation (18). Lyme arthritis can be treated with non-steroidal anti-inflammatory drugs (NSAIDs), hydroxychloroquine, or synovectomy to control inflammation of joints. However, these treatments cause many long-term toxic side effects, are not always successful, and could result in a treatment-refractory state.

Acupuncture has been used by millions of people worldwide to treat pain and inflammation during various ailments, but the effects and its mechanism are just being unraveled (19, 20). Electroacupuncture (EA) is a minimally invasive strategy for nerve stimulation to control inflammation. The vagus nerve has been shown to control inflammation in multiple experimental conditions (21–23). Our previous studies indicated that ST36 stimulation can control the immune responses to bacterial infections, which led us to predict that such stimulation could also module inflammatory responses during Lyme disease (19, 24, 25). From a clinical perspective, ST36 stimulation is the most common treatment in acupuncture for inflammatory and infectious disorders (20). Selective stimulation of the sciatic–vagal network with EA at the ST36 Zusanli acupoint modulates the production of dopamine, which mediates anti-inflammatory activity (24). Conversely, dopamine agonists mimic EA and attenuate inflammation in the experimental sepsis model (24). The ST36 acupoint in mice is located at the anterior tibial muscle 5 mm lateral to and below the anterior tubercle of the tibia. ST36 stimulation attenuates systemic inflammatory responses to bacterial infection (24, 26).

EA has been employed by researchers to modulate inflammatory responses to bacterial infections and cause analgesic effects in various animal model systems including collagen-induced rheumatoid and experimental arthritis or adjuvant-induced arthritis in rats (27–34). No scientific study has been conducted until now to elucidate the effects of EA on Lyme arthritis. Previous studies have shown that EA stimulates dopamine production (24, 35, 36). An increase in dopamine, norepinephrine, and epinephrine significantly affects sciatic–vagal nerve stimulation through a mechanism involving adrenal glands (37). Furthermore, by including inhibitor reserpine, dopamine production was demonstrated to be critical for the anti-inflammatory effects of EA (24, 36, 38). In this study, we have elucidated EA as an alternative treatment approach to alleviate arthritis and inflammation in susceptible C3H/HeJ mice infected with a bioluminescent strain of *B. burgdorferi* N40. If successful, as an alternative to expensive anti-cytokine therapy or immunosuppressive drugs with detrimental side effects, EA in combination with antibiotic therapy could be an important approach to treat persistent Lyme disease.

## Materials and Methods

### Culture of *Borrelia burgdorferi*

Bioluminescent *B. burgdorferi* N40 strain carrying a firefly luciferase gene (Bbluc) (hereafter referred to as N40) was used in this study (39) and was cultured at 33°C in Barbour-Stoenner-Kelly-II (BSK-II) medium supplemented with 6% rabbit serum. The spirochetes were harvested and adjusted to 10^4^ per 100 µl of media to use as the inoculum dose per mouse.

### Animal Work and Ethics Statement

All work with *B. burgdorferi* was conducted in the BSL2 Biosafety cabinet using precautions as approved by Rutgers Institutional Biosafety Committee. The Institutional Animal Care and Use Committee (IACUC) members reviewed and approved the protocol of the corresponding author to conduct this study at Rutgers New Jersey Medical School following guidelines of the Animal Welfare Act, The Institute of Laboratory Animal Resources Guide for the Care and Use of Laboratory Animals, and the Public Health Service Policy that are fully adopted at the Rutgers University. C3H/HeJ mice were purchased from Jackson Laboratories (West Grove, PA, USA) at 3 weeks of age to use in the experiments in this study 1 week later after they were acclimatized. The animals were housed in the institutional animal facility in micro isolator cages, and procedures were carried out in sterile environments in a biosafety cabinet.

### Mouse Infection and Electroacupuncture Treatment

C3H/HeJ mice were divided into two groups; the control (mock-treated) and EA-treated groups with 6–10 mice per group, and all were infected with 10^4^ *B. burgdorferi* (unless mentioned otherwise) on day 1 on the lateral aspect of the right thigh. Prior to EA or mock treatment, mice were anesthetized with 100 µl of a 100 mg/ml of 1:1 ketamine and xylazine combination, and physical restraint was achieved by strapping them with tape on a dissecting board. Treatments were carried out on days 0, 1, 2, 3, 4, 5, 7, 8, 9, 11, and 13 (**Supplementary Figure 1**). Stainless steel 12-mm unipolar needle electrodes (EL452; Biopac Systems, Goleta, CA, USA; **Supplementary Figure 2**) were inserted approximately 3 mm deep at the ST36 Zusanli acupoint. The ST36 Zusanli point is located 2 mm lateral to the anterior tubercle of the tibia in the anterior tibial muscle and 4 mm distal to the knee, lower joint (40). EA and subsequent nerve activation were achieved by continuous electrical stimulation of a potential difference of 4 V administered on both sides simultaneously. A continuous 4-V AC of 40 mA, a pulse width of the 50 s, and a frequency of 10 Hz using the electrostimulator (STM 150, Biopac Systems, Goleta, CA, USA) were controlled and monitored using the AcKnowledgment Lab Assistant GLP software (ACK100W-G, Biopac Systems, Goleta, CA, USA) (24) for 10 min per mouse after which mice were allowed to recover. Control mice were subjected to the same anesthetic and restraint method, but EA needles were replaced with non-conductive plastic needles applied on the skin at the acupuncture points for 10 min/mouse. Mice were monitored for signs of distress throughout the course of the experiment. Similar Lyme arthritis severity has been observed in both sexes of mice infected with *B. burgdorferi* N40 strain; however, we repeated the experiment once in male mice to include sex as a biological variable for EA treatment. The joints of mice under anesthesia were measured using a digital caliper (Fisher Scientific, Waltham, MA, USA; Cat. No. 15-077-958). Live imaging of mice was conducted as previously described (39) on days 14 and 28 post-infection (p.i.) using an IVIS-200 machine (Perkin Elmer, Waltham, MA, USA), and after euthanasia, heart, joints, bladder, and spleen were recovered. To recover live spirochetes to ascertain disseminated infection, injection site skin, ear, skin from the neck, bladder, and one kidney and 5 drops of blood were used to inoculate BSK+RS medium, and the presence of motile *B. burgdorferi* observed by dark-filed microscopy (**Supplementary Figure 1**).

### Sample Analysis

Blood was collected by cardiac puncture from anesthetized mice before euthanasia and sera recovered. Spleen and heart from each mouse were homogenized in cell lytic solution (Sigma, St. Louis, MO, USA; #C3228) with protease inhibitors and were then subjected to centrifugation at 12,000 ×*g* for 10 min. The supernatants were immediately stored at −80°C until analysis for the cytokine concentration in these extracts and sera by LEGENDplex (BioLegend, San Diego, CA, USA) according to the manufacturer’s instructions. Histopathological analysis was performed to evaluate the effect of EA on inflammatory responses in the knee and tibiotarsus joints. Specific qPCR was also performed on DNA isolated from half section of the heart and one joint of each mouse (41).

### Histopathology and Fluorescence Immunohistochemistry of Joints and Heart

One joint of each infected mouse was fixed in 4% paraformaldehyde. Joint samples were first incubated with EDTA, embedded in paraffin, then sectioned into 5–6-µm-thick slices, and mounted on microscope slides. For histopathology, joint sections from five mice were stained with H&E using standard conditions. Destruction of articular cartilage, lymphocytic infiltration, and synovial hyperplasia was recorded for joint inflammation in sections. For fluorescence immunohistochemistry, TruBond 380 slides (Newcomer Supply, Middleton, WI, USA) were used for mounting and samples processed from five mice. For antigen retrieval for fluorescence immunohistochemistry, slides with joint sections were left in the vacuum chamber overnight at room temperature followed by deparaffinization by 2 changes of xylene (5 min each) the next day. Slides were then hydrated using 2 changes of 100% ethanol (3 min each), 95% ethanol (1 min), 90% ethanol (1 min), and 80% ethanol (1 min) before being rinsed with distilled water. The slides were then heated in sodium citrate buffer at 65°C/100°C for 10 min and then stored at 4°C in phosphate-buffered saline (PBS). For immunolabeling, the samples were first blocked with 1% donkey serum-containing PBS for 30 min at room temperature and then incubated with primary antibodies against CD39, CxCR3, and CCR6 (anti-CD39 sheep polyclonal, Thermo Fisher Scientific PA5-47624; anti-CCR6-rat MAB590, Bio-Techne Corporation, Minneapolis, MN, USA; and anti-CxCR3-rabbit, Thermo Fisher Scientific PA5-23104) at 1:200 dilution in PBS containing 1% horse serum and 1% bovine serum albumin (BSA) at 37°C for 5 h followed by overnight incubation at 4°C. The slides were washed 3 times for 5 min each with PBS and then incubated with the corresponding secondary antibodies conjugated with fluorophores (Alexa Fluor 488-conjugated donkey anti-sheep antibodies from Abcam (Cambridge, UK) ab 150177, Alexa Fluor 647-conjugated chicken anti-rat antibodies from Molecular Probes/Life Technologies (Carlsbad, CA, USA) A11070, and Alexa Fluor 568-conjugated donkey anti-rabbit antibodies from Thermo Fisher Technologies A10042). For leukocyte staining in the joints, sections were incubated with 1:200 dilution of anti-mouse Ly6G rabbit antibodies (Abcam ab238132) for neutrophil staining for 5 h at 37°C. After the slides were washed with PBS three times, 10 min each time, 1:200 dilution of anti-rabbit TRITC antibodies (Thermo Fisher 16101), 1:100 dilution of anti-F4/80-APC-conjugated antibodies for macrophage (Thermo Fisher 17-4801-82), and 1:60 dilution of anti-CD3-Alexa Fluor 488-conjugated antibodies (R&D Systems, Minneapolis, MN, USA; FAB4841G) were added; slides were incubated for 5 h at 37°C. After being washed 3 times, 10 min each with PBS, slides were mounted with coverslips using an antifade mounting medium. Immunoreactivity was visualized using a confocal fluorescence microscope.

### Statistical Analysis

All data collected were analyzed by GraphPad Prism, version 7, for Windows (GraphPad Software, San Diego, CA, USA) with horizontal lines depicting medians in the dot-plots, and potential outliers are also included in the figures. Throughout the manuscript, each dot in the figure represents an individual mouse evaluated in the given experimental setup. By the Shapiro–Wilk test, the normal distribution has been validated. Pearson’s test was used to compare the correlations between treated and untreated groups, with p-values below 0.05 indicating a strong correlation. The unpaired two-samples t-test was used to compare the means of two independent groups, mock- and EA-treated. Since one group of mice was EA-treated, we considered the effect of this treatment on cellular proliferation, activation, and cytokine production independent of mock-treated mice. The results have also been verified with the Wilcoxon test for median comparison, which provided similar results. Comparisons between two groups were made using an unpaired, two-tailed Student t-test with unequal variance with differences with p < 0.05 considered significant for comparison at a 95% CI.

## Results

### Electroacupuncture Treatment of C3H/HeJ Mice does not Affect *Borrelia burgdorferi* Burden in Organs

**Figure 1:**
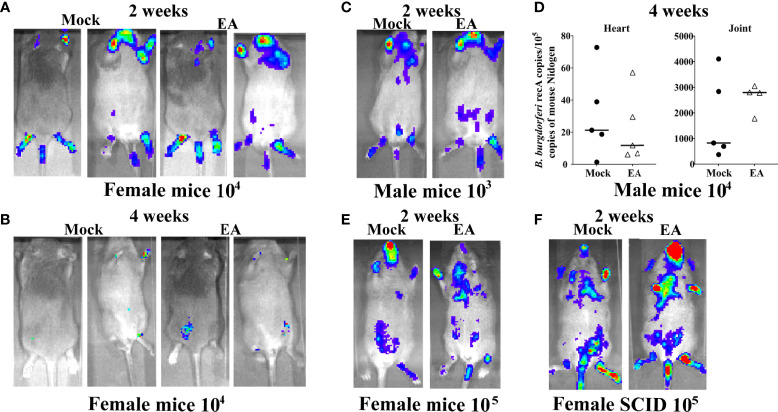
Colonization of organs of 4-week-old C3H/HeJ mice by *Borrelia burgdorferi* N40 strain did not change after EA treatment. **(A, B)** Real-time images of a representative female C3H/HeJ infected mouse (10 mice for each treatment) using IVIS-200 displayed bioluminescence as a semi-quantitative indicator of *B. burgdorferi* colonization in different organs of mice at 2 **(A)** and 4 weeks **(B)** post-infection. Spirochete burden was comparable in mock- and EA-treated mice. **(C)** Overall, light emission in representative male C3H/HeJ mouse, representing colonization of organs and spirochetal burden at 2 weeks post-infection, was not different from that observed in female mice **(A)** and was comparable in mock- and EA-treated mice. **(D)** Determination of burden of *B. burgdorferi* by real-time qPCR confirmed results obtained by live imaging **(C)**, such that mock- and EA-treated mice showed similar levels of heart and joint colonization of 5 mice each. Bars indicate median values. **(E)** Increasing infection dose to 10^5^ *B. burgdorferi*/mouse did not appreciably increase over colonization levels of organs in mice infected by 10^4^ spirochetes/mouse. **(F)** SCID mice showed higher colonization of organs by *B. burgdorferi* (higher bioluminescence radiance) than immunocompetent mice **(A–E)**; however, spirochete burden was comparable in mock- versus EA-treated mice. EA, electroacupuncture; SCID, severe combined immunodeficient.

The schedule and experiment plan are depicted in **Supplementary Figure 1**. After subcutaneous injection of *B. burgdorferi*, the spirochetes disseminated and colonized the hearts, joints, brains, etc., causing mild to severe inflammation and pathology. Spirochete burden in different organs of mice was compared between EA- and mock-treated control animals at 2 and 4 weeks p.i. by live imaging using IVIS-200. Images from both dorsal and ventral sides indicated that EA treatment did not affect the spirochete burden (**Figure 1A**). At 4 weeks, mice cleared *B. burgdorferi* significantly with barely detectable bioluminescence recorded (**Figure 1B**). Therefore, one representative mouse from 3 additional experiments at only 2 weeks p.i. is shown (**Figures 1C–F**). We observed that results were similar in male and female mice regardless of the dose of inoculum used at 2 weeks (**Figures 1A, C, E**) and 4 weeks p.i. (not shown). After euthanasia at 4 weeks p.i., various tissues were collected. Spirochete burden assessment by bioluminescence radiance levels was confirmed by qPCR of *recA* gene in hearts and joints of male infected mice (5 mice each) (**Figure 1D**). These results agree with our previous findings that radiance levels by bioluminescent N40 strain are reflective of the spirochete burden assessed by qPCR (42). To determine if the lack of adaptive immune response and missing mature T and B lymphocytes result in higher spirochete burden and arthritis, we examined severe combined immunodeficient (SCID) mice and found that bioluminescence radiance reflecting spirochete burden was similar in mock- and EA-treated mice, albeit the overall colonization level was higher in these mice (**Figure 1F**) as compared to immunocompetent C3H mice. Live spirochetes were recovered from all tissues examined by growing in a *B. burgdorferi* medium (data not shown).

### Electroacupuncture Attenuates Joint Swelling and Lyme Arthritis in C3H/HeJ Immunocompetent and Severe Combined Immunodeficient Mice

**Figure 2:**
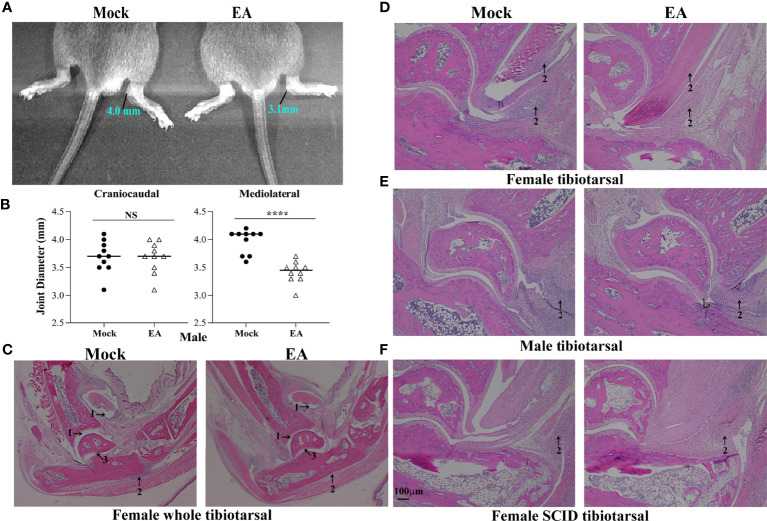
Attenuation of joint inflammation and arthritis in EA-treated mice. **(A, B)** Mediolateral and not craniocaudal measurements of tibiotarsus joints of 10 mice showed a significant reduction in swelling after EA treatment. Bars indicate median values. **(C–F)** Severe arthritis in tibiotarsus joint manifested by an increase in synovial space (arrow 1), higher leukocyte infiltration (blue stain, arrow 2), and synovial hyperplasia and erosion of cartilage (arrow 3) in composite tibiotarsus image **(C)** were observed in mock-treated *Borrelia burgdorferi*-infected mice. Infiltration of leukocytes as depicted by blue-stained dots reduced in EA-treated mice consistently. Bar in panel F represents a size of 100 µm. EA, electroacupuncture.

Arthritic manifestations are visually apparent when C3H mice are infected with our N40 strain. To determine the effect of EA on inflammation and joint swelling caused by *B. burgdorferi*, the joint diameter was determined by a vernier caliper for 10 mice for each treatment. Measurements of each joint were made from lateral to medial, and cranial to caudal, and average values were determined. One month after infection, control mice showed higher joint swelling than EA-treated mice even though EA treatment was stopped at 2 weeks p.i. (**Figures 2A, B**). Mediolateral measurements, and not craniocaudal joint measurement, indicated that EA treatment mitigates *B. burgdorferi*-stimulated joint swelling significantly. We further examined H&E-stained joint sections using a light microscope. Microscopic examination of joint sections by two trained Veterinarians in a blinded manner showed higher infiltration of leukocytes, as marked by arrows, increasing overall inflammation in mock- compared to EA-treated mice. Although the change in synovial space, synovial hyperplasia, and cartilage erosion was clearly observed in some (**Figure 2C**) but not all (**Figures 2D, E**) immunocompetent mice, leukocyte infiltration and joint swelling were reduced in all EA-treated mice. Resolution of synovial space expansion and cartilage erosion in some control mice could be attributed to overall diminished spirochete burden at 4 weeks of infection and efficient repair of cartilage in these still young infected mice. Significant infiltration of leukocytes in tibiotarsus joints was independent of the sex of mice, was noticed in immunocompetent as well as SCID female mice, and was associated with joint swelling in mock-treated mice. Infiltration of leukocytes in the joints of SCID mice suggested that intrusion of cells involved in innate immunity, such as neutrophils and to a lesser extent macrophages, is a major player associated with Lyme arthritis in both groups of mice (**Figure 2F**), while T lymphocytes could also be involved in inflammatory processes in immunocompetent hosts. Thus, the overall reduction of leukocyte infiltration induced by EA may diminish the severity of inflammatory Lyme arthritis in both sets of mice.

### Alteration in Population of T Cells in Joints After Electroacupuncture Treatment in C3H/HeJ Mice

**Figure 3:**
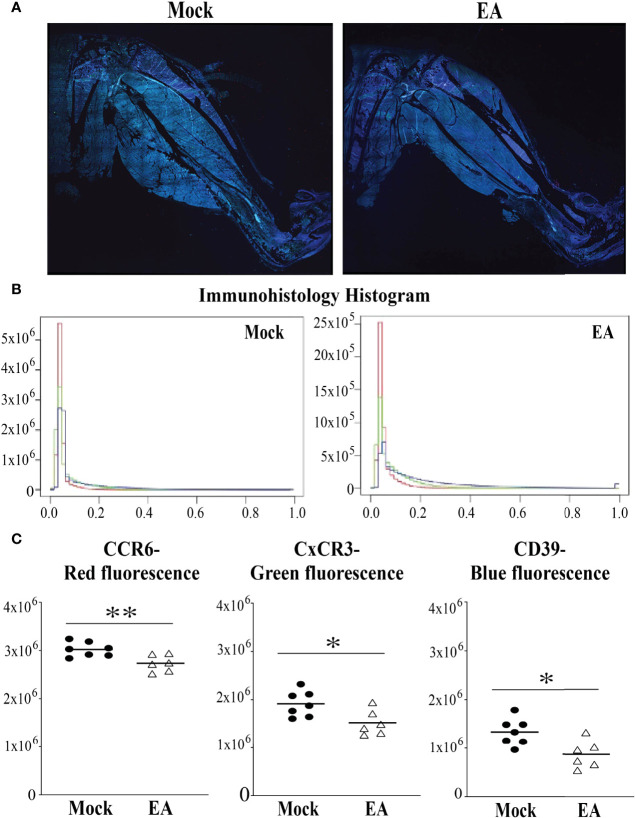
Fluorescence immunohistopathological examination of male mice joints to determine the effect of EA on CD4+ Th17 (CCR6 staining), Th1 (CxCR3 staining), and Tregs (CD39 staining) infiltration. **(A–C)** Both Th1 and Th17 cell infiltration diminished in whole joint after EA treatment significantly. Treg levels in EA-treated mice appear to be sufficient, albeit reduced, to counterbalance the effect of Th17 and Th1 in causing inflammatory Lyme arthritis. All three fluorescence signal pixels were counted using the microscope and are presented here (B,C). Statistical analysis was conducted using two-tailed unpaired Student’s t-tests for unequal variance to determine significant differences between the paired groups (NS, not significant; *p < 0.05, **p < 0.01). EA, electroacupuncture.

We examined the whole limb by immunohistochemistry to determine the impact of EA at the point where the most pronounced inflammatory arthritic symptoms are observed. Thus, to determine the infiltration of specific T cells in the joints and evaluate the effect of EA on these cells, we examined the level of CD4+ Th1 (CxCR3), Th17 (CCR6), and Treg cells (CD39) by fluorescence immunohistopathology of joint sections. Interestingly, all three cells were reduced in EA-treated mice (**Figure 3**). As a peripheral tissue trafficking marker, the CCR6 marker is also associated with activated memory like Treg cells, and a higher frequency of CCR6-positive Treg cells was reported previously in the joints of rheumatoid arthritis patients and in inflamed joints of a collagen-induced arthritis mouse model. Although a reduced level of CD39 labeling was observed in EA-treated mice, activated Treg cells in the joints still appear to be sufficient to counteract the inflammatory response by the remaining Th1 and Th17 cells. Thus, the memory anti-inflammatory role of Tregs in the joints of EA-treated mice could facilitate the reduction of Lyme arthritis severity. Diminished inflammatory response and joint swelling in these mice therefore could be due to an overall net reduction in inflammatory cytokines in the joints after EA.

### Systemic Effect of Electroacupuncture on Proinflammatory Cytokine Production in C3H/HeJ Mice

**Figure 4:**
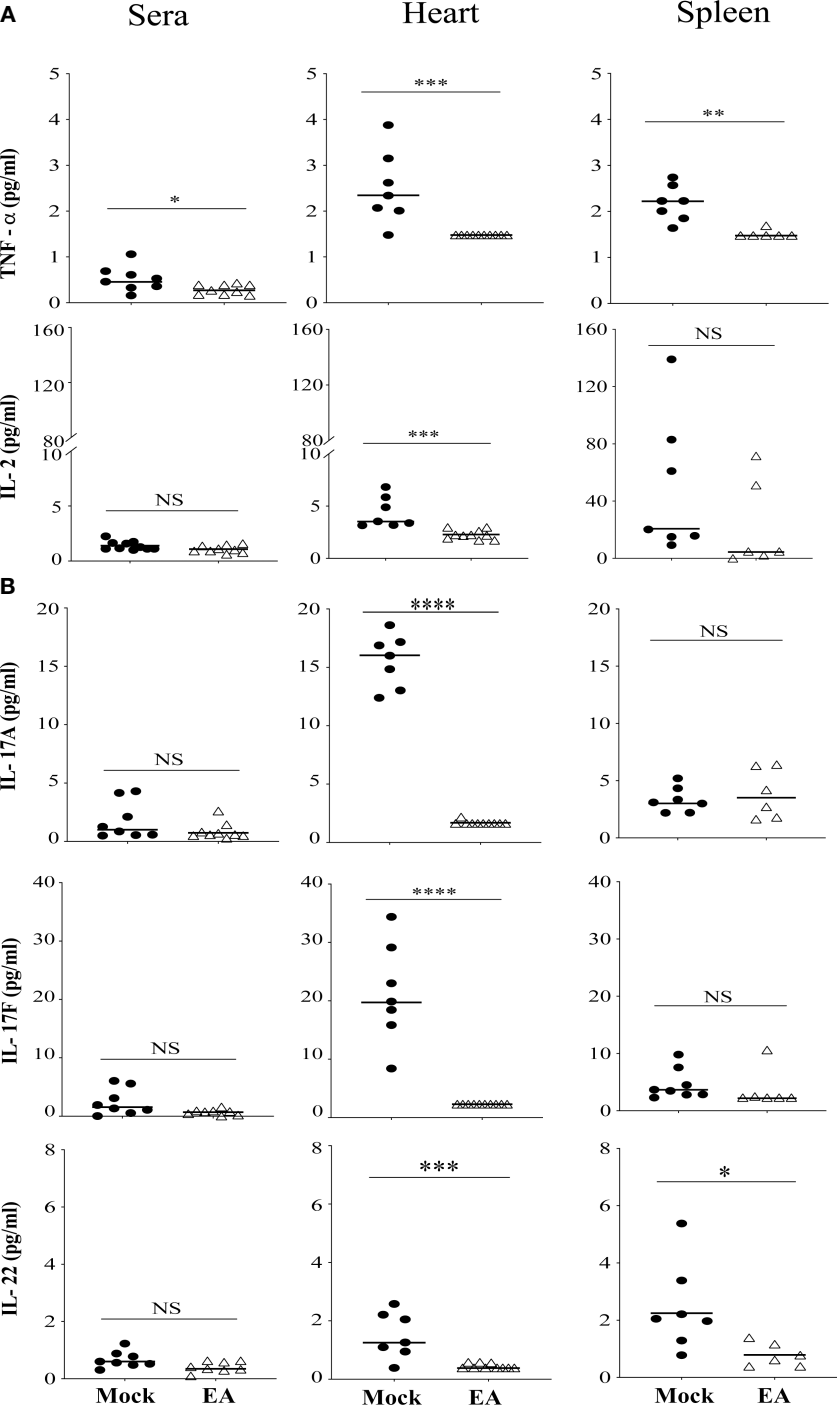
Systemic effect of EA on proinflammatory cytokines levels in serum and extracts from heart and spleen of *Borrelia burgdorferi*-infected C3H/HeJ male mice. **(A)** Proinflammatory cytokine TNF-α reduced significantly after EA treatment in serum, and heart and spleen extracts, while IL-2 reduced significantly only in heart and not spleen extracts or serum. **(B)** IL-17A, IL-17F, and IL-22 cytokines produced mostly by Th17 cells were reduced significantly in heart in EA-treated mice at 4 weeks post-infection (p.i.), while spleen also showed significant reduction in IL-22 levels. Bars indicate median values. Statistical analysis was conducted using two-tailed unpaired Student’s t-tests for unequal variance to determine significant differences between the paired groups (NS, not significant; *p < 0.05, **p < 0.01, ***p < 0.001, ****p < 0.0001).

Cytokine analysis in synovial joint fluid demonstrates response at a specific time point that could be transient because of the constant flux of cytokine levels in the accumulated fluid. Therefore, to evaluate the effects of EA on systemic inflammatory responses in *B. burgdorferi*-infected mice, the levels of selected Th1, Th2, and Th17 cytokines were determined in serum, and spleen and heart extracts using a multiplex cytokine kit (LEGENDpleX # 740005 mouse Th cytokine panel, BioLegend) according to the manufacturer’s instructions. We determined cytokine levels in the spleen as an indication of B- and T-cell responses because the spleen as the major secondary lymphoid organ also drives T- and B-cell proliferation. Cytokines in the heart were measured because there is a pathophysiological connection between Lyme arthritis and myocarditis. The pathogenesis of infection with N40 is accompanied by severe inflammation in several tissues including the joints, heart, and brain. Therefore, we examined the levels of various cytokines: TNF-α and IL-2 produced by Th1 cells (**Figure 4A**) and IL-17 and IL-22 produced by Th17 cells (43) in serum, spleen, and heart extracts from the mock- and EA-treated infected mice (**Figure 4B**). The most pronounced effects of EA treatment in infected mice were a significant reduction in Th17 cell-derived IL-17A, IL-17F, and IL-22 cytokines in the heart at 4 weeks p.i. (**Figure 4**). EA treatment also reduced splenic IL-22 levels significantly. Of note, EA was even more effective in reducing TNF-α and IL-2 levels in the heart and TNF-α levels in the spleen. The levels of Th1 cell-derived cytokines in sera were very low, but EA further reduced TNF-α levels in sera albeit usually not significantly. There was a strong correlation in levels of proinflammatory cytokines, TNF-α, IL-2, IL-17A, IL-17F, IL-22, and IFNγ, between blood and heart (R = 0.644 in mock-treated and 0.6503 in EA-treated); however, the correlation between blood and spleen was very low (below 0.05). This attests to the greater involvement of the heart during the dissemination of Lyme spirochetes. TNF-α cytokine directly impairs endothelial function by reducing nitric oxide synthase expression and triggering NF-κB activation and reactive oxygen species accumulation that increase vascularity and infiltration by polymorphonuclear cells and destruction of joint tissue. NF-κB activation causes further expression of proinflammatory cytokines. Blunting of TNF-α by EA likely generates an anti-inflammatory effect for alleviating joint and other organ inflammation.

### Th1 and Th2 Cell Activation and Cytokine Production Affected by Electroacupuncture in C3H/HeJ Mice

**Figure 5:**
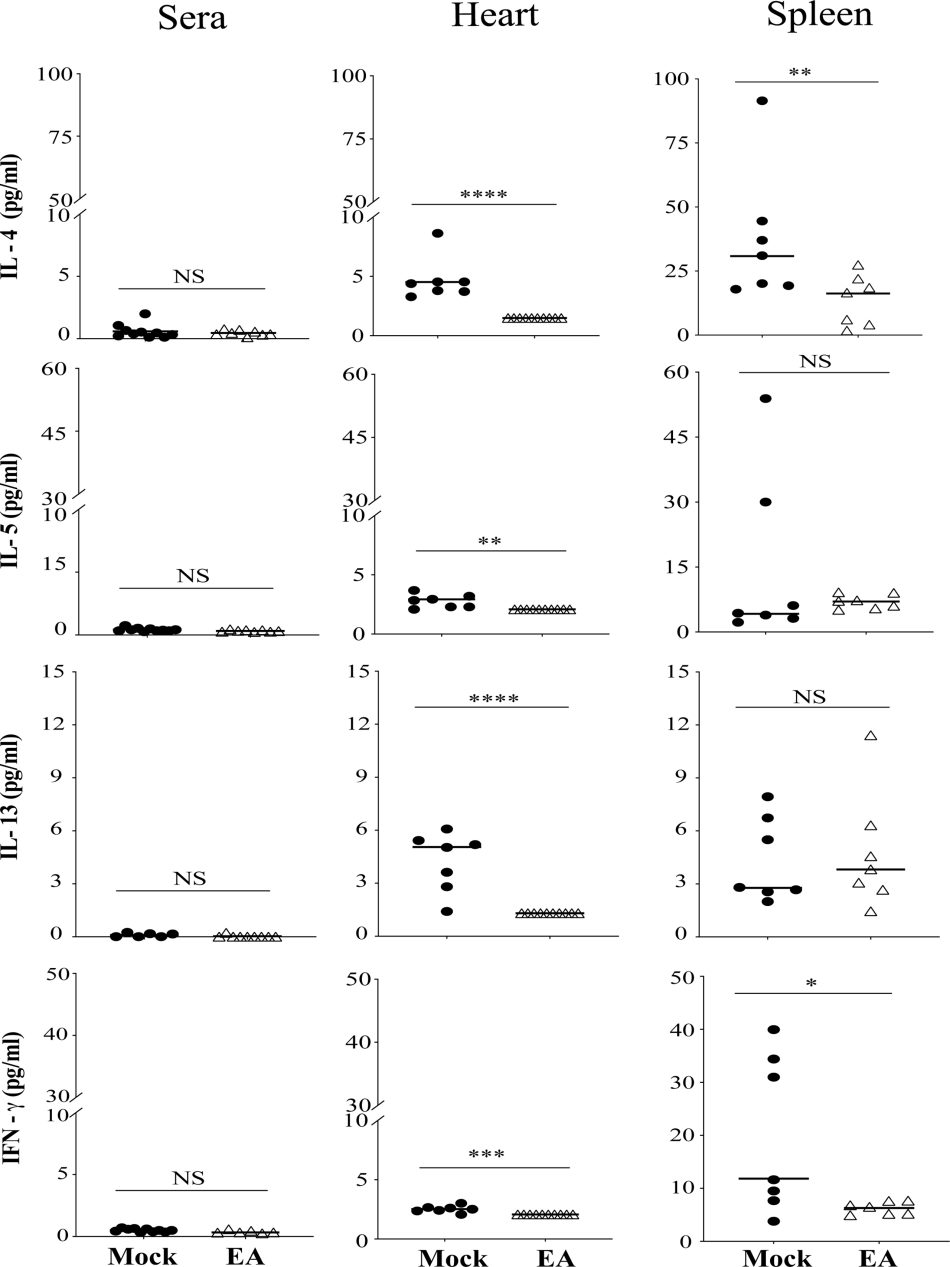
Effect of EA treatment on cytokines production by CD4+ Th1 and Th2 cells in serum, spleen, and heart extracts of *Borrelia burgdorferi*-infected C3H/HeJ male mice. IL-4, IL-5, and IL-13 cytokines that are primarily produced by Th2 cells were reduced significantly in heart after EA treatment while proinflammatory Type II IFN-γ cytokines were reduced significantly in heart as well as spleen. Overall lower levels of these cytokines were detected by LEGENDplex in EA-treated mice in this study. Bars indicate median values. Statistical analysis was conducted using two-tailed unpaired Student’s t-tests for unequal variance to determine significant difference between the paired groups (NS-Not significant, *p < 0.05, **p < 0.01, ***p < 0.001, ****p < 0.0001). EA, electroacupuncture.

We further examined the systemic effect of EA on Th1 and Th2 cells by measuring selected cytokine levels. Levels of Th2 cytokines IL-13, IL-5, and IL-4, and IFN-γ production associated with Th1 and other immune cells were determined. Th2 response is known to facilitate specific antibody production in response to infection. As a whole, Th2 responses were significantly reduced in the hearts of EA-treated mice. The effect of EA treatment on spleen and serum cytokines was only moderate. IFN-γ levels were lower in the heart at 4 weeks p.i. and further reduced in the heart and spleen of EA-treated mice significantly (**Figure 5**).

### Neutrophils are the Major Cells Contributing to Lyme Arthritis, and Electroacupuncture Reduces their Infiltration in Joints of C3H/HeJ Mice

**Figure 6:**
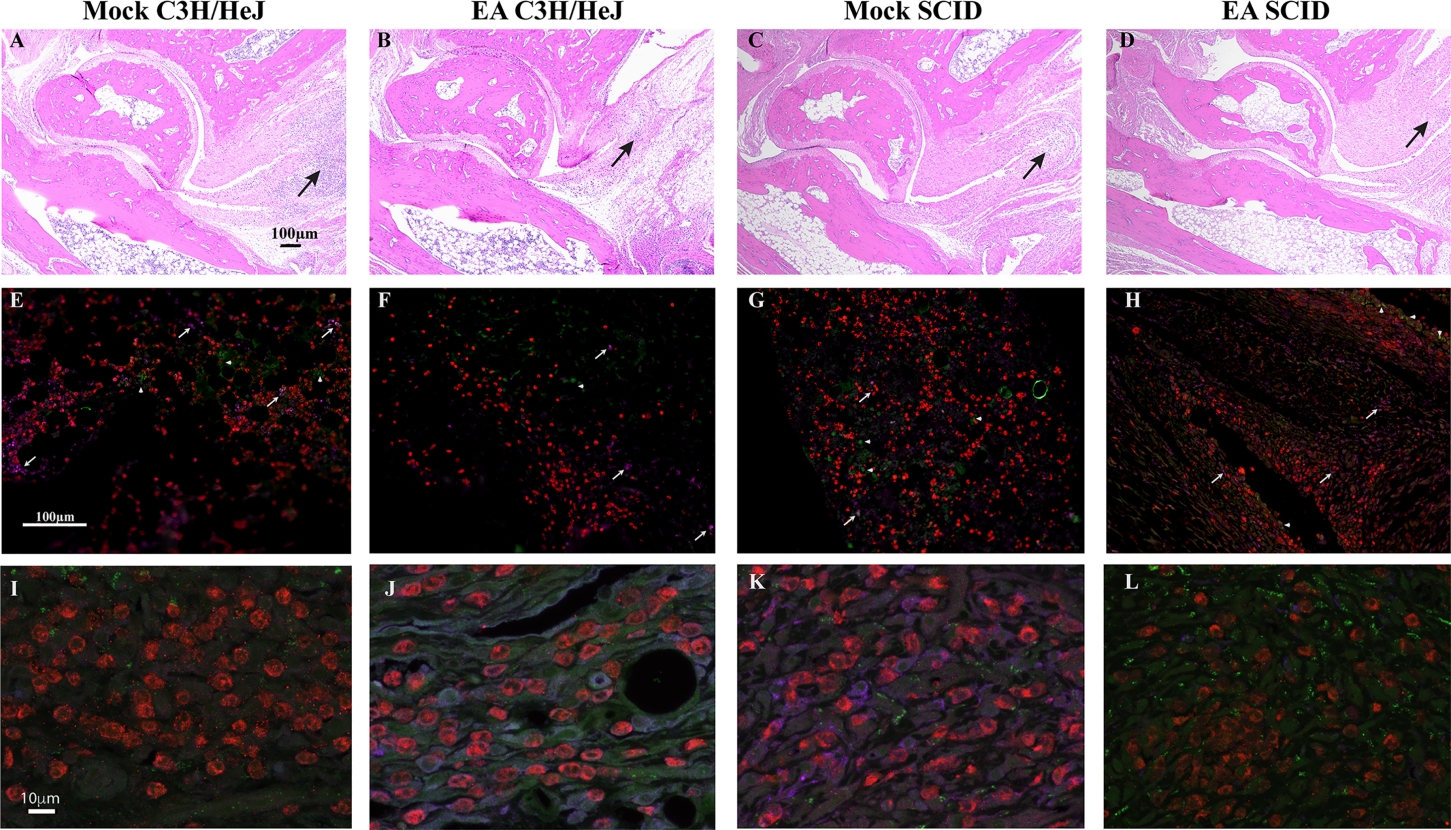
Comparative analysis of mock- and EA-treated female immunocompetent and SCID C3H female mouse joint sections to determine inflammation. **(A–D)** Leukocyte infiltration was reduced in EA-treated mice (B and D versus A and C) irrespective of immunological genotype of mice. Leukocyte infiltration (arrows) showed association with joint swelling (not shown) and diminished in the joints of EA- compared to mock-treated mice. Cartilage damage and increase in synovial space resolved in these young mice at this stage. **(E–H)** Neutrophils (red, anti-Ly6G rabbit antibodies followed by anti-rabbit TRITC-conjugated antibodies) were the predominant leukocytes in the joints of all infected mice, and their levels were reduced in EA-treated mice. Only a few macrophages in panels E–H (purple, anti-F4/80-APC antibodies, arrows) and T cells in panels E and F (green, anti-CD3-Alexa Fluor 488 antibodies, arrowheads) were observed in tibiotarsus joints. **(I–L)** Higher magnification of sections shown in panels **E–H**. Green cells in SCID mice joints **(G, H, K, L)** are likely NKT cells. EA, electroacupuncture; SCID, severe combined immunodeficient.

Flow cytometric immunophenotyping may offer sensitive detection of specific cell types; however, we instead used paraffin immunohistochemical phenotyping, which offers preservation of architecture and evaluation of an expression in the specific section of hind legs. Reduced joint swelling in EA-treated immunocompetent and SCID mice was associated with reduced infiltration of leukocytes (**Figures 6B, D**). We found that neutrophils were the predominant leukocytes in the joints with only a few macrophages (**Figures 6E-H**) and T cells observed (**Figures 6E, F**). Overall, levels of neutrophils were observed to be mock SCID > mock female > EA female = EA SCID mice. In SCID mice, cells labeled with anti-CD3 antibodies could represent NKT cells in the joints since these mice lack mature T cells. Images of infiltrated cells in mock- and EA-treated C3H/HeJ mice and C3H/HeJ SCID mice are also shown to depict joint inflammatory response and its attenuation (**Figures 6I–L**). Innate and adaptive immune responses mediated by neutrophils as well as T cells contribute to inflammatory arthritis in immunocompetent mice (**Figures 2**, **3**), while neutrophils appear to be the main contributors to joint inflammation in SCID mice, such that the resolution of inflammation in these mice is associated with reduced infiltration of neutrophils in the joints after EA treatment.

### Electroacupuncture Reduces Inflammation in Joints, Hearts, and Spleens in Mice Infected by *Borrelia Burgdorferi*

**Figure 7:**
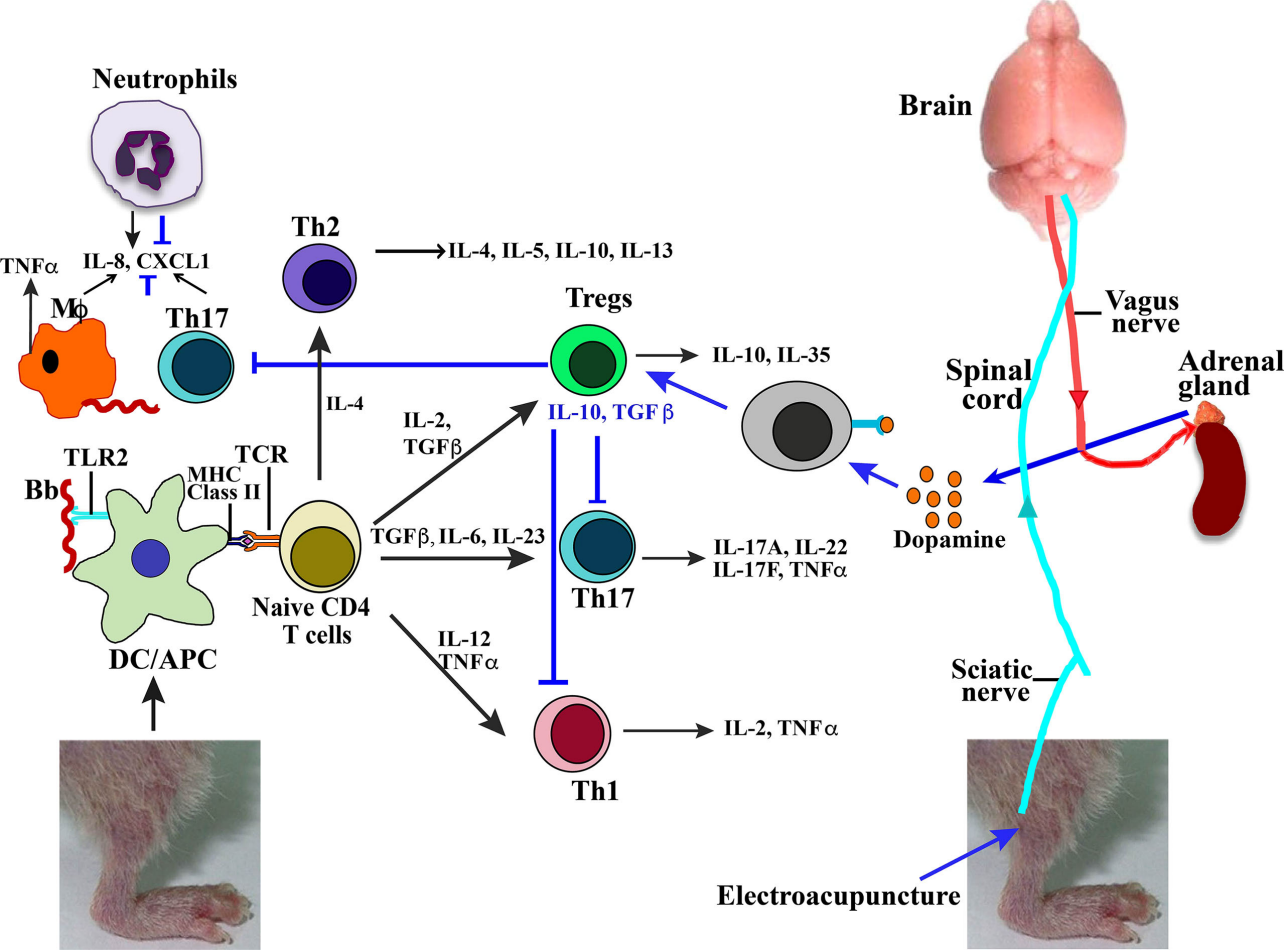
Model summarizing effect of *Borrelia burgdorferi* infection and EA treatment on joint inflammatory responses and arthritis attenuation. High concentration of lipoproteins on *B. burgdorferi* surface signal through TLR2 on antigen-presenting cells to cause naive CD4+ cells to differentiate in different types of T cells in response to various combinations of cytokines produced. These Treg, Th17, or Th1 cells then produce listed cytokines (marked by black arrows). Neuromodulation of brain through sciatic nerve electrostimulation by EA results in signaling of adrenal glands through vagus nerve to produce dopamine (marked by blue arrows). Binding of dopamine neurotransmitter to receptor present on lymphocytes results in activation of Tregs, which then suppress Th1- and Th17-mediated inflammatory Lyme arthritis. EA, electroacupuncture.

We propose a model based upon our previous studies (Akoolo 2021, Torres-Rosas 2014) and data presented here that bring together the effect of *B. burgdorferi* infection and anti-inflammatory response mechanisms involved during EA treatment of mice. Lyme spirochetes induce moderate to severe Lyme arthritis in humans and different strains of mice by triggering inflammatory response *via* TLR signaling in antigen-presenting cells including dendritic cells, mostly due to TLR2 stimulation by spirochete lipoproteins (**Figure 7**, left side). Depending on the levels and type of cytokine produced, stimulation of different immune cell types could occur, and Lyme arthritis severity may vary depending on the stimulation of cells involved in pro- versus anti-inflammatory responses. Recruitment of neutrophils by secretion of macrophage and Th17 lymphocytes (and likely others) by the production of chemokine ligand 1 (CXCL1) results in synovitis in humans and Lyme arthritis in susceptible C3H strains of mice. In fact, similar to our observation here (**Figure 6**), neutrophils have been shown to play an important role in causing inflammation during Lyme arthritis in wild-type mice, while infiltration of both CD4 and CD8 cells increases in TLR2 knockout mice (44). Furthermore, depletion of CD8 cells in TLR2^−/−^ mice lowered spirochete burden and reduced arthritis severity in mice. CXCL1 produced by macrophages and lymphocytes, especially Th17 cells, has been shown to recruit neutrophils during Lyme arthritis (45). Thus, a reduction in infiltration of macrophages and lymphocytes after EA treatment could also reduce neutrophils and inflammation in the joints (**Figure 6**).

Th17 and Th1 cells have also been implicated in Lyme arthritis such that neutralization of their impact may not be sufficient for the anti-inflammatory effects of Tregs. Human and murine lymphocytes express dopamine D2-like receptors including DRD2, DRD3, and DRD4. Thus, EA signaling through the sciatic–vagal network causes dopamine production, which could shift the balance between Treg/Th17 and reduce Th17 and Th1 cells (**Figure 7**, right to left) to alleviate Lyme arthritis. Reduction in Th17 cells, and also macrophage infiltration, could further diminish recruitment of neutrophils due to reduced CXCL1 levels, further contributing to Lyme arthritis resolution after EA treatment.

## Discussion

Lyme arthritis is a chronic inflammatory condition and is characterized by hyperplasia of the synovium, destruction of joint articular surfaces, and an excess of inflammatory cells in the periarticular tissues. This results in progressive destruction of joints as also observed in rheumatoid arthritis and thus offers a good model system to study arthritis etiology. Complex immunological mechanisms involved in causing inflammatory arthritis manifestations as presented in this study and those documented in previously published reports together with a reported response to EA treatment are summarized in **Figure 7**.

Infection by *B. burgdorferi* often causes severe inflammation in the joints, heart, and brain among other tissues (46). Many patients exhibit persistent musculoskeletal pain, chronic fatigue, and cognitive dysfunction long after completion of the antibiotic treatment regimen for Lyme disease, often defined as PTLDS. Accurate diagnosis of PTLDS is complicated by the employment of two-tier serological tests that detect antibodies against *B. burgdorferi* even after spirochetes are cleared. To some extent, posttreatment Lyme arthritis in individuals could be attributed to dysregulated immune responses due to both the slow release of spirochete peptidoglycan and triggered autoimmunity as indicated by detection of high levels of proinflammatory cytokines and chemokines in the synovial fluid of such patients (47). Therefore, the successful treatment of late Lyme disease could be facilitated by a combination of both antimicrobial and anti-inflammatory therapeutic approaches.

Susceptible animal models have contributed significantly to understanding the mechanisms of Lyme arthritis in humans. We have used information obtained from previously published literature and data presented in this study to propose a model of Lyme arthritis mechanisms (15, 27–29, 32, 45, 48), inflammatory responses to infectious diseases, or PAMPs as affected by EA treatment (19, 20, 37, 49, 50), and to suggest relevance to human Lyme disease as summarized in **Figure 7**. Recruitment of neutrophils by secretion of macrophage and Th17 lymphocytes (and likely others) by the production of CXCL1 results in synovitis in humans and Lyme arthritis in susceptible C3H strains of mice (**Figure 7**). In fact, neutrophils have been shown to play an important role in causing inflammation during Lyme arthritis in immunocompetent mice, while infiltration of both CD4 and CD8 cells increases in TLR2 knockout mice (44). Furthermore, depletion of CD8 cells in TLR2^−/−^ mice has been reported to lower spirochete burden, causing reduced arthritis severity in mice. CXCL1 produced by macrophages and lymphocytes, especially Th17 cells, has been shown to recruit neutrophils during Lyme arthritis (45). Thus, a reduction in infiltration of macrophages and lymphocytes after EA treatment could also reduce neutrophils and inflammation in the joints.

The interaction between the nervous and immune systems plays a major but often overlooked role in regulating processes required for maintaining physiological homeostasis and in responding to acute stressors like infections in the body (51). Neuronal control has been used in arresting inflammatory processes that accompany infectious diseases (52). Our study unveils a therapeutic application of communication between the nervous and immune systems to curb inflammatory responses to *B. burgdorferi* infections (**Figure 7**). In this investigation, we exploited the stimulation of the sciatic–vagal network with EA to alleviate inflammatory Lyme arthritis in susceptible young infected C3H/HeJ mice. The recruitment of leukocytes into sites of inflammation is crucial for the pathogenesis of arthritis and other inflammatory conditions. In arthritis, several Th1 and Th17 cytokines are involved in almost all aspects of articular inflammation and destruction (53, 54). The onset of Lyme arthritis is closely related to the T helper cells and the associated Th1, Th2, and Th17 cytokines. Control of inflammation is therefore directed predominantly to innate (primarily neutrophils) and adaptive Th1 and Th17 cell immune responses. EA has been shown to upregulate the levels of dopamine in murine models (55). Murine and human lymphocytes express dopamine D2-like receptors, and previous studies have shown that activation of these receptors diminishes inflammatory arthritic manifestations caused by an imbalance in Th17/Treg cells (15). TGFβ cytokine plays a critical role in maintaining the balance of the Th17/Treg cells such that TGFβ with IL-6 or IL-23 promotes differentiation of Th17 cells, while TGFβ with IL-2 induces differentiation of Tregs (16, 17). Furthermore, TLR2 stimulation by *B. burgdorferi* lipoproteins can also activate these cells’ proliferation (18, 48). We found that EA reduced the splenic levels of critical inflammatory factors such as TNF-α in *B. burgdorferi*-infected mice. Treatment of arthritis in mice with antibodies against these proinflammatory cytokines TNF-α and IL-6 has been shown to attenuate the disease (55). The net adaptive inflammatory response even in autoimmune arthritis depends on the balance between proinflammatory cytokines produced by Th1 and Th17 cells that are counteracted by Treg cell response.

Previous studies in patients with rheumatoid arthritis and juvenile idiopathic arthritis in an animal model have reported the presence of T cells expressing CXCR3 and CCR6 in synovial tissues (56). Further studies have elucidated that CXCR3 plays a non-redundant role in recruiting inflammatory T cells in arthritis (57). Blockade of CXCR3 has also been observed to reduce inflammatory cell infiltration and attenuate arthritis (58). Increased expression of CD39 has also been observed in patients with arthritis; increased CD39 expression was seen on synovial mononuclear cells (59, 60). IL-17-producing Th17 cells have also been shown to predominantly express CCR6 receptors in an animal model of rheumatoid arthritis (61). Immunohistochemical staining of serial sections of joints showed that Th1 (CxCR3 stained) and Tregs (CD39 surface marker) levels were significantly reduced in EA-treated mice, while Th17 (CCR6) were also reduced. Although infection-induced arthritis could be different from autoimmunity-associated arthritis (62), the effectiveness of CxCR3 antagonist in reducing collagen induced inflammatory arthritis in mice supports our data (63).

IL-22 is important in epithelial skin barriers, therefore playing a role in the dissemination of the organism (64). Although no difference in dissemination was observed, there was increased joint swelling in mock as compared to EA-treated mice due to the anti-inflammatory action of sciatic–vagal network stimulation. There is growing evidence that IL-17 and/or IFN-γ levels are elevated in samples from human and mouse Lyme arthritis. Th17/Th1 response has been seen to contribute to the persistent inflammation with a significant role played by IL-17F, IL-17A, and IL-22. To evaluate the systemic response of EA through neuronal signaling, a panel of cytokines was examined in the heart and spleen of infected mice (**Figures 4**, **5**). TLR2-mediated stimulation of T cells together with this treatment restored the normal balance of Th1-Th17/Tregs and alleviated Lyme arthritis for a prolonged period of time (24, 35, 36). EA apparently mitigated the inflammatory role of IL-17 in Lyme arthritis. An antagonistic role has been documented between IL-17 and IL-10 in Lyme borreliosis, and the role of IL-10 in Borrelia infection has been defined as aiding in dissemination. We did not observe any difference in disseminated infection or immediate effect of EA on tissue colonization by N40 by live imaging and qPCR. We recovered live *B. burgdorferi* from the bladder, skin, and/or blood of all infected mice (data not shown), which is also an indicator of colonization by Lyme spirochetes. Our model summarizes previously documented effects of *B. burgdorferi* infection on immune responses in wild-type mouse joints causing inflammatory Lyme arthritis (**Figure 7**). Based upon the data presented in this study, we have also summarized the specific and systemic inhibitory effects of EA on Lyme spirochete-induced inflammatory responses and on reduction in infiltration of total leukocytes, particularly neutrophils in the joints, to cause decreased joint swelling and inflammatory Lyme arthritis resolution.

This is the first study to investigate and evaluate the positive effects of EA treatment on Lyme arthritis. Inflammation is often associated with the clearance of infecting pathogen; however, excessive inflammatory responses lead to tissue damage and need to be controlled to attenuate disease manifestations (19, 49, 50). Suppression of the host’s immune system to reduce inflammation can also impede the elimination of pathogens. Our results agree with previous findings that the nervous control of inflammation occurs when the sciatic–vagal network is activated.

The limitation of this study is that although EA mitigates the effects of inflammation in rodents, it has not been evaluated in humans to treat various types of arthritic manifestations. Anti-inflammatory responses of EA could be advantageous as an alternative treatment approach for patients without introducing the adverse side effects of immunosuppressive steroids or needing highly expensive treatment involving antagonists of proinflammatory cytokines. However, EA conditions will need to be fully optimized for human use first before using it to prevent or alleviate PTLDS or antibiotic-refractory Lyme disease symptoms. Once approved, effective EA treatment could improve the quality of life of patients with persistent Lyme disease when combined with antibiotic treatment to clear the bacterial infection.

## Data Availability Statement

Data supporting the findings of this study are included in the article/**Supplementary Material**. Further inquiries can be directed to the corresponding author.

## Ethics Statement

All work with B. burgdorferi was conducted in BSL2 Biosafety cabinet using precautions as approved by Rutgers Institutional Biosafety Committee. The Institutional Animal Care and Use Committee (IACUC) members reviewed and approved the protocol of the corresponding author to conduct this study at Rutgers New Jersey Medical School following guidelines of the Animal Welfare Act, The Institute of Laboratory Animal Resources Guide for the Care and Use of Laboratory Animals, and the Public Health Service Policy that are fully adopted at the Rutgers University.

## Author Contributions

NP designed the study and contributed to manuscript writing and editing. LA and VD performed experiments and together with SCR analyzed the data. LA wrote the initial draft of the manuscript, and LU, VD, and SCR contributed to manuscript editing. All authors read and approved the final manuscript.

## Funding

This work is supported by the National Institute of Allergy and Infectious Diseases, Award Numbers R01AI089921 and R01AI137425, to NP.

## Conflict of Interest

The authors declare that the research was conducted in the absence of any commercial or financial relationships that could be construed as a potential conflict of interest.

## Publisher’s Note

All claims expressed in this article are solely those of the authors and do not necessarily represent those of their affiliated organizations, or those of the publisher, the editors and the reviewers. Any product that may be evaluated in this article, or claim that may be made by its manufacturer, is not guaranteed or endorsed by the publisher.

## Supplementary Material

Supplementary Figure 1 Scheme of experimental plan used in this study.

Supplementary Figure 2 Image of Biopac electrostimulation system used for mock and EA treatment of *B. burgdorferi* infected mice.
