# Supplementary figures and images for: Sciatic–Vagal Nerve Stimulation by Electroacupuncture Alleviates Inflammatory Arthritis in Lyme Disease-Susceptible C3H Mice
Source: Front Immunol. 2022 Jul 18;13:930287. doi: 10.3389/fimmu.2022.930287 (PMC9342905; doi:10.3389/fimmu.2022.930287)

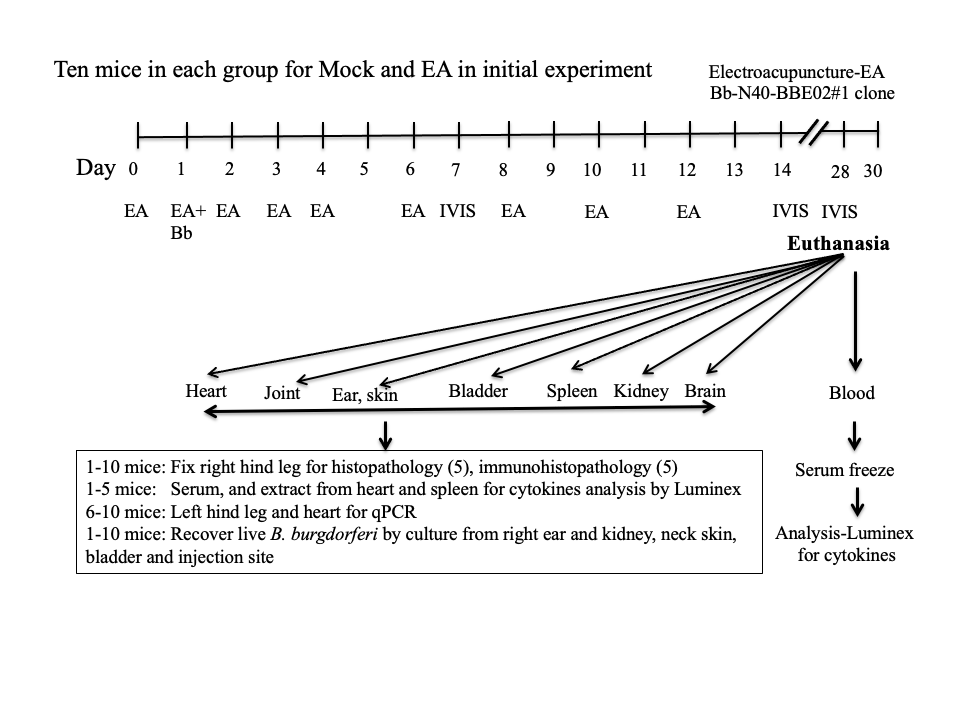

Supplement: Supplementary Figure 1 — Scheme of experimental plan used in this study. [file Image_1.tiff]

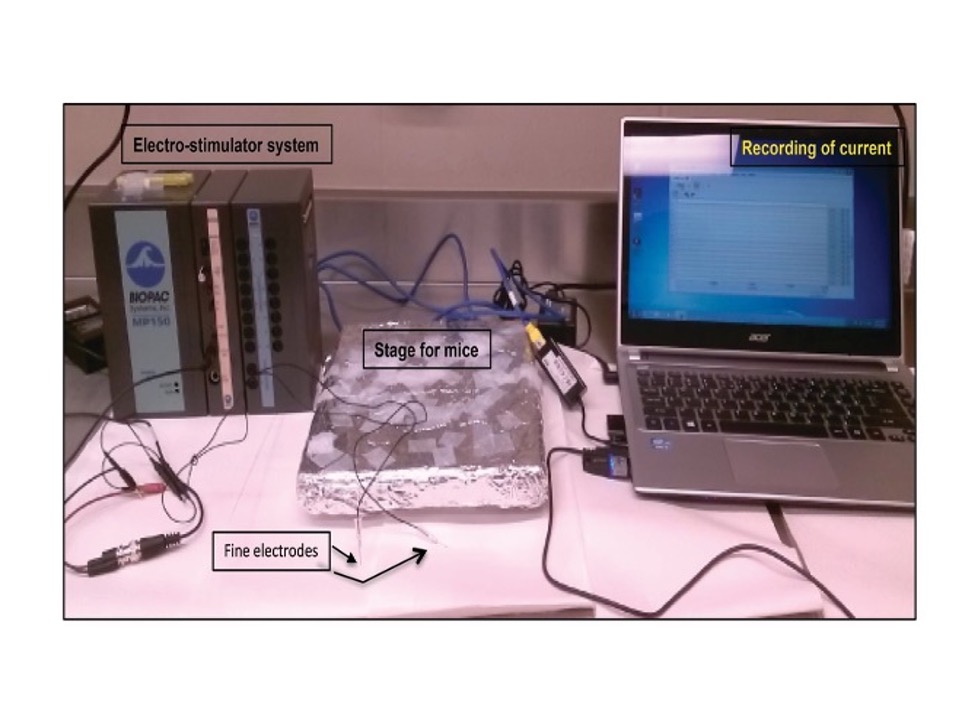

Supplement: Supplementary Figure 2 — Image of Biopac electrostimulation system used for mock and EA treatment of B. burgdorferi infected mice. [file Image_2.jpeg]
